# Separable processes for live “in-person” and live “zoom-like” faces

**DOI:** 10.1162/imag_a_00027

**Published:** 2023-11-07

**Authors:** Nan Zhao, Xian Zhang, J. Adam Noah, Mark Tiede, Joy Hirsch

**Affiliations:** Department of Psychiatry, Yale School of Medicine, New Haven, CT, United States; School of Psychology and Cognitive Science, East China Normal University, Shanghai, China; Department of Neuroscience, Yale School of Medicine, New Haven, CT, United States; Department of Comparative Medicine, Yale School of Medicine, New Haven, CT, United States; Wu Tsai Institute, Yale University, New Haven, CT, United States; Department of Medical Physics and Biomedical Engineering, University College London, London, United Kingdom

**Keywords:** interactive face encoding, zoom-like vs. in-person faces, functional near-infrared spectroscopy (fNIRS), hyperscanning, visual sensing, neural coupling

## Abstract

It has long been understood that the ventral visual stream of the human brain processes features of simulated human faces. Recently, specificity for real and interactive faces has been reported in lateral and dorsal visual streams, raising new questions regarding neural coding of interactive faces and lateral and dorsal face-processing mechanisms. We compare neural activity during two live interactive face-to-face conditions where facial features and tasks remain constant while the social contexts (in-person or on-line conditions) are varied. Current models of face processing do not predict differences in these two conditions as features do not vary. However, behavioral eye-tracking measures showed longer visual dwell times on the real face and also increased arousal as indicated by pupil diameters for the real face condition. Consistent with the behavioral findings, signal increases with functional near infrared spectroscopy, fNIRS, were observed in dorsal-parietal regions for the real faces and increased cross-brain synchrony was also found within these dorsal-parietal regions for the real In-person Face condition. Simultaneously, acquired electroencephalography, EEG, also showed increased theta power in real conditions. These neural and behavioral differences highlight the importance of natural, in-person, paradigms and social context for understanding live and interactive face processing in humans.

## 1 Introduction

Interpretation of dynamic facial cues as well as their spontaneous reciprocity during live interactions are generally considered to be essential social skills for creating meaningful social bonds and modulating social communications. The live and real expressive human face provides primary cues for natural in-person social interactions. Increased reliance on on-line webcam platforms for interpersonal face-to-face communications motivates questions of how the neural responses to virtual interactions compare to natural face-to-face interactions. The recent emergence of “Zoom-like,” i.e., webcam-mediated, face-to-face interactions as a global mode of social and transactional communication accentuates the importance of understanding face processing in natural and “everyday” environments and also in virtual on-line conditions. Comparison of “on-line” and “in-person” live face gaze introduces a novel paradigm for neuroscience questions in the “everyday world” that add insight into the neural and behavioral mechanisms of live face-to-face interactions. Based on previous findings that suggest live faces activate lateral and dorsal-parietal systems in the human brain that are not activated by simulated face stimuli (Hirsch et al., 2022; Kelley, Noah, Zhang, Scassellati, & Hirsch, 2021; Noah et al., 2020), we hypothesize that these live face-processing mechanisms will increase for in-person relative to on-line face gaze. Findings from this study will be taken as further evidence in support of the importance of real social interactions between dyads for investigations of face encoding systems in the human brain.

### 1.1 Specialization for faces

The processing of faces is typically modeled by hierarchical pathways consisting of specialized regions within the ventral stream including the fusiform face area, the lateral occipital cortex, and temporal gyri (Engell & Haxby, 2007; Haxby, Hoffman, & Gobbini, 2000; Kanwisher, McDermott, & Chun, 1997). Specialized face-processing mechanisms are commonly thought to be innate, an interpretation supported by the frequent observation of a stereotyped hierarchy of face-processing regions (Haxby, Gobbini, Furey, Ishai, & Pietrini, 2001; Haxby, Gobbini, Furey, Ishai, Schouten, et al., 2001; Ishai, Ungerleider, Martin, Schouten, & Haxby, 1999). Evidence consistent with face-selective domains in the cortex includes findings for holistic face processes including reduced sensitivity to inverted faces relative to upright faces (Diamond & Carey, 1986; Kanwisher, Tong, & Nakayama, 1998; Tanaka & Farah, 1991), and face pathways associated with social behaviors (Johnson et al., 2005). This specialized feature-based model has been referred to as a “top-down” model (Arcaro & Livingstone, 2021), and the findings are primarily based on evidence from simulated, mostly static, faces with low- to medium-level face-like features using single participant paradigms. Further, these models are based on non-interactive faces and therefore probe a limited domain of facial features that do not include dynamic and real social interaction. Although important for controlled experimental conditions, these conventional representational stimuli and paradigms do not provide information related to the functional organizations tuned to acquire and process live face-to-face interactions as they unfold in natural conditions, and therefore limit the generalizability of current face-processing models.

### 1.2 Interactive face processing and spoken language

Second-person neuroscience (Redcay & Schilbach, 2019), however, focuses on interaction-specific neural mechanisms of in-person face processing under naturalistic scenarios. Hyperscanning, simultaneous imaging of two individuals during live interactions has provided a powerful approach for investigating the neural mechanisms of social behavior (Dumas & Fairhurst, 2021; Hasson, Ghazanfar, Galantucci, Garrod, & Keysers, 2012; Hoehl, Fairhurst, & Schirmer, 2021; Montague et al., 2002). Specifically, hyperscanning allows for an examination of how each brain can influence the other during social interaction (Davidesco et al., 2023; Ellingsen et al., 2023). For example, cross-brain coherence has been found in the left inferior frontal cortex during a face-to-face dialog between partners but none during a back-to-back dialog, a face-to-face monologue, or a back-to-back monologue (Jiang et al., 2012), and simple talking and listening with interaction between dyads increased activity in left Wernicke’s Area compared to the no-interaction condition. Cross-brain coherence was also increased between these regions during the interaction conditions (Hirsch et al., 2021). Comparisons of similar tasks with Zoom formats have reported reduced conversational turn-taking behavior and cross-brain coherence compared to in-person interaction (Balters, Miller, Li, Hawthorne, & Reiss, 2023). These findings contribute to a growing body of evidence in support of neural specificity for interpersonal and live social interactions.

### 1.3 Multi-modal comparison of faces presented in-person and on Zoom-like media

The introduction of multi-modal acquisitions extends approaches to investigate the domain of live interactions. For example, simultaneous fNIRS and electroencephalographic, EEG, neuroimaging technologies support advanced interrogations of live face processing under real dyadic interactive conditions that include both spatial and temporal variables (Jiang et al., 2012; Koike, Sumiya, Nakagawa, Okazaki, & Sadato, 2019; Leong et al., 2017; Piazza et al., 2020). Simultaneous data acquired from interacting dyads enable multi-modal investigations of the underlying neurobiology of live face processing based on hemodynamic signals (Cui, Bryant, & Reiss, 2012). A theoretical framework for “two-person” face processing is grounded in the interactive brain hypothesis, which proposes that both neural and cognitive systems are altered during live interactions relative to similar behaviors performed in “solo” modes (De Jaegher, Di Paolo, & Adolphs, 2016; Di Paolo & De Jaegher, 2012). Consistent with the interactive brain hypothesis, spectral analysis of electrical brain activity using dual-brain EEG before and during visually mediated social coordination found oscillatory components that increased with coordinated behavior within the human mirror neuron system (Tognoli, Lagarde, DeGuzman, & Kelso, 2007). Further, increases in early-stage EEG processing of facial information for real, in-person eye gaze compared with eye gaze at a picture have also been reported (Pönkänen, Alhoniemi, Leppänen, & Hietanen, 2011). These EEG findings are consistent with more recent fNIRS findings that relate interactive face processing to social mechanisms (Carter & Huettel, 2013) associated with neural activity in right temporal and dorsal parietal regions of brain (Kelley et al., 2021; Noah et al., 2020). Together, both electrical and hemodynamic brain activity suggests that social interactions are mediated by specialized neural mechanisms that contribute a theoretical framework for a new “neuroscience of two” (Redcay & Schilbach, 2019; Schilbach et al., 2013). Observation of differences between in-person and virtual on-line presentations of the same live faces in this investigation would be taken as further evidence in support of the importance of naturalistic conditions for live and interactive face processing.

Current models of face processing do not predict differences between conditions where the facial features do not vary. Here, we test the specific hypothesis that social context (real and in-person vs. real and on-line) will increase measures of variables that contribute to real and in-person face processing relative to the on-line conditions. These measures include behavioral eye tracking and visual dwell times on the face (Schroeder, Wilson, Radman, Scharfman, & Lakatos, 2010) as well as arousal as indicated by pupil diameters (Beatty, 1982). Similarly, neural signals acquired by fNIRS in dorsal-parietal and lateral regions of interest would be expected to increase for the In-person condition if social cues were enhanced consistent with prior measures of live vs. simulated faces (Hirsch et al., 2022; Noah et al., 2020). These regions have also been associated with salience detection and visual guidance (Braddick, Atkinson, & Wattam-Bell, 2003; Gottlieb, Kusunoki, & Goldberg, 1998), and would predict increased coherence for the live-In-person condition due to the additional salience of a physically present partner. Finally, simultaneously acquired event related potentials (ERP) have been implicated in processing of facial features (Bentin, Allison, Puce, Perez, & McCarthy, 1996; Dubal, Foucher, Jouvent, & Nadel, 2011; Itier & Taylor, 2004; Pönkänen et al., 2011); and are not expected to differ in this experiment because the face features are common to both conditions. However, increases in theta power activity have been reported for cognitive and attentional processes (Ptak, Schnider, & Fellrath, 2017) as well as for processes associated with facial expressions (G. G. Knyazev, Slobodskoj-Plusnin, & Bocharov, 2009; Zhang, Wang, Luo, & Luo, 2012), and to the extent that cognitive, attentional, and expressive cues are enhanced during In-person conditions, an increase in theta power is expected.

## 2 Materials and Methods

**Fig. 1.**
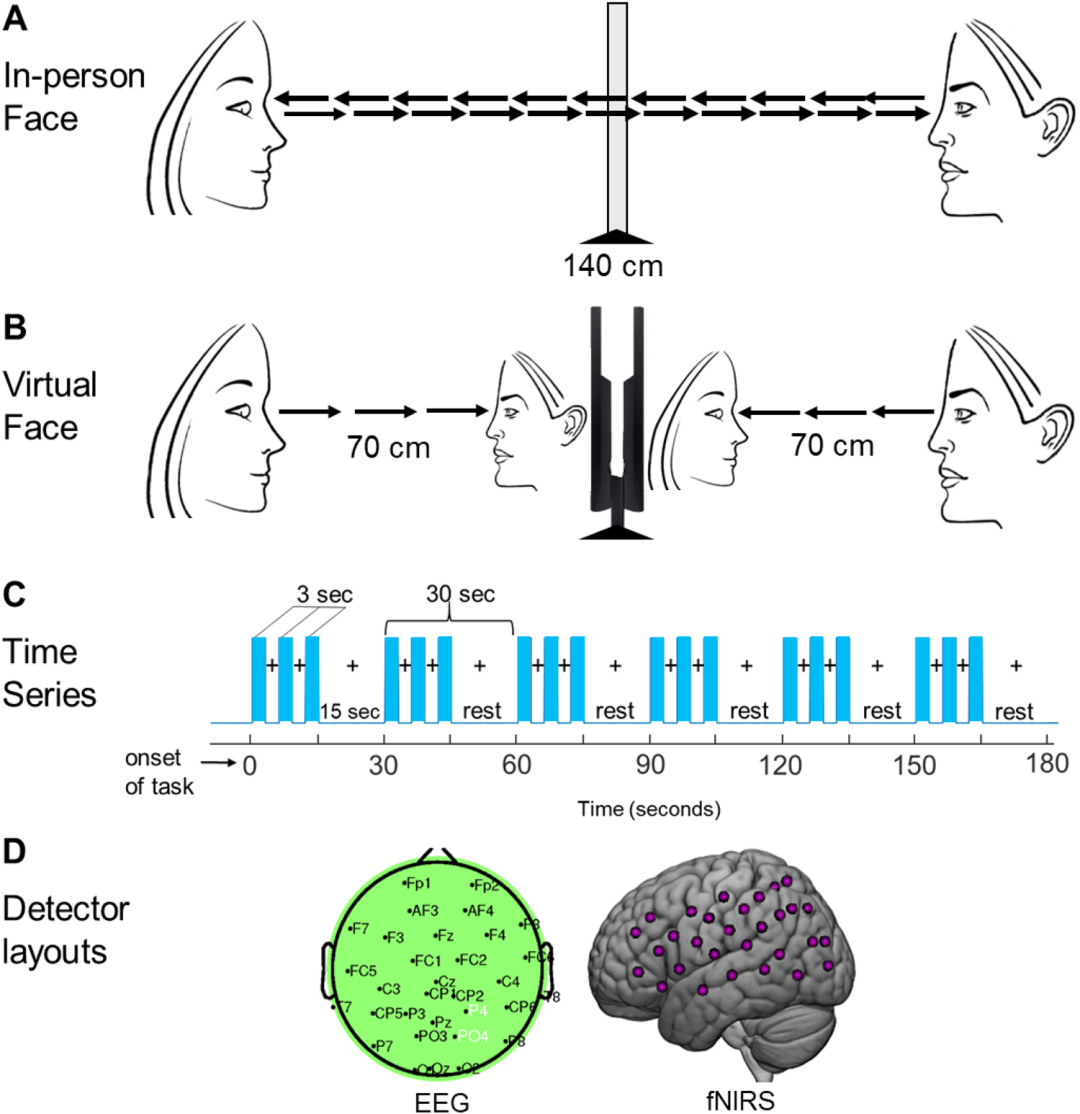
Experimental conditions and time series. (A) In-person Face condition. Partners were seated across from each other separated by 140 cm with a glass panel placed between them at the midpoint (70 cm) that alternated between transparent and opaque. (B) Virtual Face condition. Two 24-inch 16 × 9 monitors were placed between the participants at a viewing distance of 70 cm and matched to subtend the same visual angle as the real face. Each participant watched their partner’s face on a monitor in real time as their images were transmitted via cameras located above the monitors. (C) Time course of the experimental paradigm. The duration of every run was 3 minutes, and each run was repeated twice for both the Virtual Face and In-person Face conditions. Each run included six alternating 15 s task and rest periods. In the task period (blue bars), participants watched their partner either on a monitor (Virtual Face condition) or through transparent smart glass (In-person Face condition) in 3-s periods alternating with 3-s periods of a blank screen (Virtual Face) or opaque glass (In-person Face). During the 15-s rest period, participants looked at a crosshair on a monitor (Virtual Face) or straight ahead at opaque smart glass (In-person Face). (D) EEG electrode placements (left) and fNIRS (right) optode placements. P4 (Extrastriate Visual Cortex, Area V3) and PO4 (Supramarginal Gyrus and Somatosensory Association Cortex) are shown in white; fNIRS channels are indicated as pink dots. Locations are included in Supplementary Tables S1a and S1b.

Dyads faced each from across a table at a distance of 140 cm and table-mounted eye-tracking systems were positioned to measure continuous and synchronized eye movements simultaneously on both partners. Functional NIRS and EEG data were also synchronized and continuously acquired hemodynamic and electrocortical responses during the experiment on both participants. For the In-person condition, dyads were separated by a “smart glass” in the center of the table that controlled face viewing times (the glass was transparent during viewing periods) and “rest times” (the glass was opaque during rest periods) (Fig. 1A). For the virtual Webcam condition the configuration was the same except that the smart glass in the center of the table was replaced by a monitor that displayed the real time face of the partner as in a Zoom-like condition (Fig. 1B). In both conditions, face viewing times were controlled according to the time series as illustrated in Figure 1C. Each experimental run was 3 minutes in duration and consisted of six task epochs each 18 s in duration and six interleaved rest epochs each 12 s in duration. Each task epoch was subdivided into three 6-s cycles of “on” and “off” face viewing. The face viewing events were 3 s of each cycle as indicated by the blue vertical bars in Figure 1C. Participants were instructed to gaze at the face and eyes of their partner whenever the face was visible and to focus straight ahead when the face was not visible. Participants were instructed not to talk during the experimental runs and to avoid sudden and large movements. Prior to starting the experiment, both partners were fit with a cap populated with optodes to acquire fNIRS data and embedded with electrodes to acquire simultaneous EEG data as illustrated in Figure 1D. Anatomical locations of the fNIRS channels and the EEG electrodes are provided in Supplementary Tables S1a and S1b, respectively. The neural and eye-tracking data streams were acquired simultaneously and also synchronized by the time series (Fig. 1C) for integrated processing. See Section 2 for further details.

### 2.1 Participants

Participants included 28 typically developed healthy adults (61% female; mean age 28.4 ± 9.8 years; 93% right-handed (Oldfield, 1971) with self-reported normal or corrected-to-normal vision. See biographical information on Supplementary Tables S2 and S3. Sample size was determined by a power analysis based on prior face gaze experiments (Noah et al., 2020) where peak brain activations between task and rest in the right temporal parietal junction, rTPJ, were t = 0.00055 ± 0.0003 (one-sided) and the distance (signal difference/standard deviation) was 0.534. Using the “pwr” package of R statistical software (Champely, 2020) at a significance of p ≤ 0.05, the sample must include 23 participants to assure the conventional power of 0.80. Our sample size of 28 meets and exceeds that standard for fNIRS investigations. Conventional sample sizes for dual-brain electroencephalogram studies are typically less than this (Pönkänen et al., 2011; Tognoli et al., 2007), and sample size was determined by the highest requirement.

All participants provided written informed consent in accordance with guidelines approved by the Yale University Human Investigation Committee (HIC # 1501015178). Dyads were assigned in order of recruitment, and participants were either both strangers prior to the experiment or casually acquainted as classmates. Participants were not stratified further by affiliation or dyad gender mix. Six pairs were mixed gender, six pairs were female-female, and two pairs were male-male.

### 2.2 Paradigm

Each dyad participated in two tasks in which they were seated 140 cm across a table from each other. In both tasks, dyads were instructed to gaze at the eyes of their partner (Fig. 1). In the In-person condition, dyads had a direct face-to-face view of each other. A “Smart Glass” (glass that is capable of alternating its appearance between opaque and transparent upon application of an appropriate voltage) panel was positioned in the middle of the table 70 cm away from each participant (Fig. 1A). In the Virtual Face condition, each dyad watched their partner’s faces projected in real time on separate 24-inch 16 × 9 computer monitors placed in front of the glass (Fig. 1B). The order of these conditions was counterbalanced. The In-person and the Virtual conditions were performed in the same location by the same dyads (see illustration in Fig. 1A and B). Participants were instructed to minimize head movements, remain as still as possible during the task by avoiding large motions, and maintain facial expressions that were as neutral as possible. The time series (Fig. 1C) and experimental details are similar to previous studies (Hirsch, Zhang, Noah, & Ono, 2017; Noah et al., 2020). At the start of a block, prompted by an auditory beep, dyads were fixated on a crosshair located in the center of the monitor in the Virtual Face condition or in the center of the opaque smart glass in the In-person condition. The face of the Virtual partner was visual-angle corrected to the same size as the In-person Face (Fig. 1B). The auditory tone also cued viewing the crosshair during the rest/baseline condition according to the protocol time series (Fig. 1C).

Six 18-s active task periods alternated with a 12-s rest/baseline period for a total of 3 minutes per run. The task period consisted of three 6-s cycles in which face presentation alternated “on” for 3 s and “off” for 3 s for each of three events (Fig. 1C). The smart glass became transparent during the “on” period and opaque during the “off” and rest periods. The time series was performed in the same way for all conditions. During the 12-s rest/baseline period, participants focused on the fixation crosshair, as in the case of the 3-s “off” periods that separated the eye contact and gaze events and were instructed to “clear their minds” during this break. The 3-s time “on” period was selected due to increasing discomfort when maintaining eye contact with a live partner for periods longer than 3 s (Hirsch et al., 2017; Noah et al., 2020). Each 3-minute run was repeated twice. The whole paradigm lasted 18 minutes. Stimulus presentation, eye-tracking data acquisition, fNIRS signal acquisition, and EEG signal acquisition were synchronized using TTL (Transistor-to-Transistor Logic) and network broadcast protocols referred to as UDP to generate triggers (details below) that were sent to all machines simultaneously.

### 2.3 Data acquisition

#### 2.3.1 Eye tracking

Eye-tracking data were acquired using two Tobii Pro x3-120 eye trackers (Tobii Pro, Stockholm, Sweden), one per participant, at a sampling rate of 120 Hz. In the In-person condition, eye trackers were mounted on the smart glass facing each participant. Calibration was performed using three points on their partner’s face prior to the start of the experiment. The partner was instructed to stay still and look straight ahead while the participant was told to look first at the partner’s right eye, then left eye, then the tip of the chin. In the Virtual Face condition, eye trackers were mounted on the lower edge of the computer monitor facing each participant, and the same three-point calibration approach was applied using the partner’s face displayed on the computer monitor via webcam.

Tobii Pro Lab software (Tobii Pro, Stockholm, Sweden) and OpenFace (Baltrušaitis, Robinson, & Morency, 2016) were used to create areas of interest for subsequent eye-tracking analyses performed in MATLAB 2019a (Mathworks, Natick, MA). UDP signals were used to synchronize the triggers from the stimulus presentation program to a custom virtual keyboard interpretation tool written in Python sent to the Tobii Pro Lab software. When a face-watching trial started and ended, UDP triggers were sent via Ethernet from the paradigm computer to the eye-tracking computers, and the virtual keyboard “typed” a letter that marked the events in the eye-tracking data recorded in Tobii Pro Lab subsequently used to delimit face-watching intervals.

#### 2.3.2 Pupillometry

Pupil diameter measures were acquired using the Tobii Pro Lab software and post-processing triggers to partition time sequences into face-watching intervals. Left and right pupil diameters were averaged for each frame and interpolated to 120 Hz as gaze position sampling.

#### 2.3.3 Electroencephalography (EEG)

A g.USBamp (g.tec medical engineering GmbH, Austria) system with 2 bio-amplifiers and 32 electrodes per subject were used to collect EEG data at a sampling rate of 256 Hz. Electrodes were arranged in a layout similar to the 10-10 system; however, exact positioning was limited by the location of the electrode holders, which were held rigid between the optode holders. Electrodes were placed as closely as possible to the following positions: Fp1, Fp2, AF3, AF4, F7, F3, Fz, F4, F8, PC5, PC1, PC2, PC6, T7, C3, Cz, C4, T8, CP5, CP1, CP2, CP6, P7, P3, Pz, P4, P8, PO3, PO4, O1, Oz, and O2. Conductive gel was applied to each electrode to reduce resistance by ensuring contact between the electrodes and the scalp. As gel was applied, data were visualized using a bandpass filter to allow frequencies between 1 and 60 Hz. The ground electrode was placed on the forehead between AF3 and AF4, and an ear clip was used for reference.

#### 2.3.4 Functional near-infrared spectroscopy (fNIRS)

A Shimadzu LABNIRS system (Shimadzu Corp., Kyoto, Japan) was used to collect fNIRS data at a sampling rate of 123 ms (8.13 Hz). Each emitter transmitted three wavelengths of light, 780, 805, and 830 nm, and each detector measured the amount of light that was not absorbed. The amount of light absorbed by the blood was converted to concentrations of OxyHb and deOxyHb using the Beer-Lambert equation. Custom-made caps with interspersed optode and electrode holders were used to acquire concurrent fNIRS and EEG signals (Shimadzu Corp., Kyoto, Japan). The distance between optodes was 2.75 cm or 3 cm, respectively, for participants with head circumferences less than 56.5 cm or greater than 56.5 cm. Caps were placed such that the most anterior midline optode holder was ≈2.0 cm above nasion, and the most posterior and inferior midline optode holder was on or below inion. Optodes consisting of 40 emitters and 40 detectors were placed on each participant to cover bilateral frontal, temporal, and parietal areas (Fig. 1D), providing a total of 60 acquisition channels per participant. A lighted fiber-optic probe (Daiso, Hiroshima, Japan) was used to remove hair from the optode channel before optodes were placed. To ensure acceptable signal-to-noise ratios, resistance was measured for each channel prior to recording. Adjustments were made until all optodes were calibrated and able to sense known quantities of light from each laser wavelength (Noah et al., 2015; Ono et al., 2014; Tachibana, Noah, Bronner, Ono, & Onozuka, 2011).

After the experiment, a Polhemus Patriot digitizer (Polhemus, Colchester, Vermont) was used to record the position of EEG electrodes and fNIRS optodes, as well as five anatomical locations (nasion, inion, Cz, left tragus, and right tragus) for each participant (Eggebrecht et al., 2012, 2014; Ferradal, Eggebrecht, Hassanpour, Snyder, & Culver, 2014; Okamoto & Dan, 2005; Singh, Okamoto, Dan, Jurcak, & Dan, 2005). Montreal Neurological Institute (MNI) coordinates (Mazziotta et al., 2001) for each channel were obtained using NIRS-SPM software (Ye, Tak, Jang, Jung, & Jang, 2009). Anatomical correlates were estimated with the TD-ICBM152 atlas using WFU PickAtlas (Maldjian, Laurienti, & Burdette, 2004; Maldjian, Laurienti, Kraft, & Burdette, 2003).

### 2.4 Data analysis

#### 2.4.1 Signal processing of eye-tracking data and calculation of duration of gaze on faces

Eye-tracking data were exported from the Tobii Pro Lab software to the data processing pipeline, and custom scripts in MATLAB were used to calculate the duration of gaze on faces, variability of gaze, and pupil diameter. OpenFace (Baltrušaitis et al., 2016) was used to generate the convex hull of an “average face” using 16 (8 pairs) of the individual OpenFace results from the Tobii videos to partition gaze directed at the face or not.

#### 2.4.2 Statistical analysis of eye contact

The gaze task alternated between eye gaze (participants were expected to fixate on the eyes of their partner’s virtual face or the eyes of their live partner) and rest (participants were expected to fixate on either the crosshair on the computer monitor [Virtual Face condition] or a red dot on the smart glass [In-person condition]). The eye gaze portions of the task were 3 s in length, and 3 epochs during each of the 18 s task blocks (Fig. 1C). Usable eye-tracking data were acquired for 20 participants (10 dyads). To avoid possible transition effects caused by shifting eye gaze between stimuli (partner’s eyes) and fixation, the initial 1000 ms of each eye gaze trial was excluded from analysis. Samples marked by Tobii as “invalid” and samples outside of the polygon defined by the average “face” by OpenFace were also discarded. Measures derived for each trial included Dwell Time (DT), computed as the number of retained samples over the gaze interval normalized by sampling rate (seconds), which represents the duration of gaze contacts on either the virtual face or the face of the live partner. To measure the variability of the gaze on the partner’s face, standard deviations were calculated by computing the log horizontal (HSD) and vertical (VSD) deviations from the mean-centered samples of each gaze interval normalized by the number of retained samples. Pupil diameter over face-watching intervals was z-scored by participant (PDZ). Linear mixed-effects models (Bates, Sarkar, Bates, & Matrix, 2007) were fitted in R (R Core Team, 2018) on DT, HSD, VSD, and PDZ separately.

#### 2.4.3 Electroencephalography (EEG)

EEG signals were preprocessed using EEGLAB v13.5.4b in MATLAB 2014a (Mathworks, Natick, Massachusetts). EEG was digitized at a sampling rate of 256 Hz. MATLAB was used to filter the data with a bandwidth of 1-50 Hz for each participant. Two types of channels exhibiting noise characteristics of poor contact with the scalp were rejected based on visual inspection: (1) signals with amplitude exceeding 100 μV, and (2) signals that were completely flat with low-frequency drift. With these criteria, an average of 3 channels per person were removed, and signals from the surrounding channels were interpolated. A common average reference was computed using the 32 data channels and averaged to produce 1 epoch data file per condition with -100 to 3000 ms epochs, where the 0 ms point is locked to face presentation (In-person Face vs. Virtual Face). The 100 ms prior to task onset served as baseline. These files were manually inspected for epochs containing eye movements and blinks, which were discarded from further analysis. The runica algorithm (Delorme, Sejnowski, & Makeig, 2007) implemented within EEGLAB was used to remove independent components associated with eye movements (blinks and left-right components). An additional IC was occasionally used to remove temporally sparse frequency abnormalities. Wavelet decomposition algorithms were applied to EEG signals within the first 250 ms to calculate the EEG power in the following frequency bands: theta (4-8 Hz), alpha (8-13 Hz), and beta (13-30 Hz). The EEG signals were decomposed into frequency components using wavelet decomposition. Unlike the FFT algorithm, the wavelet approach offers higher temporal resolution for analyzing signal events in short time periods. On the other hand, each component is associated with a range of frequencies rather than a single frequency, and therefore, it is more tolerant of frequency variation. Unlike an event-related analysis, the wavelet approach does not require events to occur at the exact same times, thus it is preferable for our paradigm of free face viewing where spontaneous micro-events are not pinned to a specific and known moment of time. Statistical comparisons based on t-tests were conducted for each frequency band.

#### 2.4.4 Functional near-infrared spectroscopy (fNIRS)

The analysis methods used here have been described previously (Dravida, Noah, Zhang, & Hirsch, 2018; Hirsch, Noah, Zhang, Dravida, & Ono, 2018; Noah, Dravida, Zhang, Yahil, & Hirsch, 2017; Noah et al., 2015; Piva, Zhang, Noah, Chang, & Hirsch, 2017; Zhang, Noah, Dravida, & Hirsch, 2017; Zhang, Noah, & Hirsch, 2016) and are briefly summarized below. First, wavelet detrending was applied to the combined (HbDiff) hemoglobin signal (the sum of the oxyhemoglobin and the inverted deoxyhemoglobin signals) (Tachtsidis et al., 2009) to remove baseline drift using the algorithm provided by NIRS-SPM (Ye et al., 2009). The combined OxyHb and deOxyHb signals are reported here, representing the most comprehensive measurement. However, consistent with best practices for fNIRS data (Yücel et al., 2021), results from the separate signals are included in Supplementary Figures S1-S2 and Supplementary Tables S5-S6. Results are generally comparable to those reported here, although reduced activity is apparent in the deOxyHb analysis due to expected factors such as noise and relative difficulty with signal detection. Second, noisy channels were removed automatically if the root mean square of the signal was more than 10 times the average for that participant. A principal component analysis spatial filter was used to remove global components caused by systemic effects assumed to be non-neural in origin (Zhang, Noah, Dravida, & Hirsch, 2020; Zhang et al., 2016, 2017). For each run, a general linear model (GLM) computed by convolving the eye gaze task paradigm (Fig. 1C) with a canonical hemodynamic response function was used to generate beta values for each channel. Group results based on these beta values were rendered on a standard MNI brain template (Fig. 4). Second-level analyses were performed using t-tests in SPM8. Anatomical correlates were estimated with the TD-ICBM152 T1 brain atlas using WFU PickAtlas (Maldjian et al., 2003, 2004).

#### 2.4.5 Wavelet coherence

Coherence analyses were performed on the combined HbDiff signals as described above in Section 2.4.4. Details on this method have been validated (Zhang et al., 2020) and applied to prior two-person interactive investigations (Hirsch et al., 2017, 2018; Piva et al., 2017). Briefly, channels were grouped into 12 anatomical regions including: (1) angular gyrus (BA39); (2) dorsolateral prefrontal cortex (BA9); (3) dorsolateral prefrontal cortex (BA46); (4) pars triangularis (BA45); (5) supramarginal gyrus (BA40); (6) fusiform gyrus (BA37); (7) middle temporal gyrus (BA21); (8) superior temporal gyrus (BA22); (9) somatosensory cortex (BA1, 2, and 3); (10) premotor and supplementary motor cortex (BA6); (11) subcentral area (BA43); and (12) frontopolar cortex (BA10) and automatically assigning the channels to these groups. The wavelet coherence analysis decomposes time-varying signals into their frequency components. Here, the wavelet kernel used was a complex Gaussian (“Cgau2”) provided in MATLAB. The residual signal from the entire data trace was used, with the activity due to the task removed, similar to traditional Psychophysiological Interaction (PPI) analysis (Friston et al., 1997). Sixteen scales were used, and the range of frequencies was 0.1 to 0.025 Hz. Based on prior work, we restricted the wavelengths used to those that reflect fluctuations in the range of the hemodynamic response function. Coherence results in the range higher than 0.1 Hz have been shown to be due to non-neural physiologic components (Nozawa, Sasaki, Sakaki, Yokoyama, & Kawashima, 2016; Zhang et al., 2020). Complex coherence values were averaged in accordance with previously established methods (Zhang et al., 2020). A total of 11 wavelengths were used incrementing from 2.475 s in steps of 2.475 s up to 27.2 s in wavelengths. Cross-brain coherence is the correlation between the corresponding frequency components across interacting partners, averaged across all time points and represented as a function of the wavelength of the frequency components (Hirsch et al., 2017, 2018; Noah et al., 2020; Zhang et al., 2020). The difference in coherence between the In-person Face and Virtual Face conditions for dyads was measured using t-tests for each frequency component. Only wavelengths shorter than 30 s were considered as the experimental cycle between task and rest was 30 s. An analysis on shuffled pairs of participants was conducted in order to confirm that the reported coherence was specific to the pair interaction and not due to engagement in a similar task. The coherence analysis was a region of interest analysis targeting somatosensory association cortices in the dorsal visual stream.

## 3 Results

### 3.1 Behavioral measures of visual sensing and pupil diameter

**Fig. 2.**
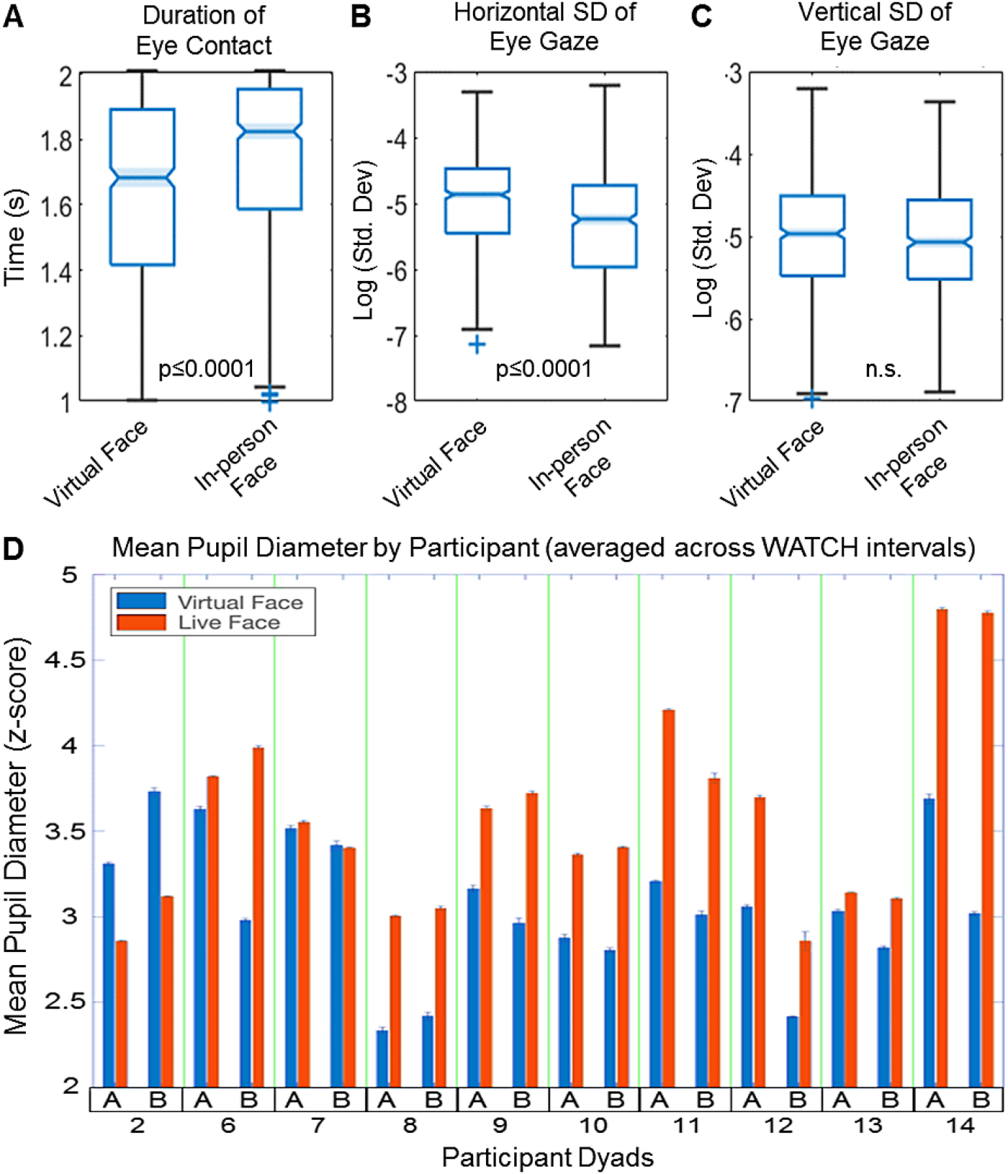
Eye contact. (A) Duration of eye contact was higher for the In-person Face condition relative to the Virtual Face condition (t = 4.01, p ≤ 0.0001). (B) Log Horizontal standard deviation of eye gaze trajectory normalized by duration of eye contact was greater for Virtual Face than In-person Face (t = -6.90, p ≤ 0.0001). (C) Log Vertical standard deviation of gaze trajectory normalized by duration of eye contact showed no difference (t = 0.15, n.s.). (D) z-Scored mean pupil diameter over viewing intervals for participant A and B within each dyad (x-axis) shows generally larger values (t = 3.81, p ≤ 0.002) for In-person Face (red) than for Virtual Face (blue).

Average gaze dwell time (DT) on the partner’s face was increased in the live In-person Face condition relative to the Virtual Face condition (t = 4.01, p ≤ 0.0001), shown in Figure 2A. Positional variance as indexed by log horizontal standard deviation normalized by DT also increased for the live condition (t = -6.9, p ≤ 0.0001), as depicted in Figure 2B. No significant differences were detected in log vertical s.d., as illustrated in Figure 2C. z-Scored mean pupil diameter across viewing epochs is shown for each participant (A, B) within the dyad. Of the 14 dyads in the study, complete sets of pupillary data were successfully acquired on 10, as depicted in Figure 2D. Mean pupil diameter was generally higher in the In-person condition (red bars) (t = 3.81, p ≤ 0.002), and within pairs partners tended to track the magnitude of each other’s responses, including an instance in PAIR 2, where mean pupil diameter *declined* in the live condition for both partners. A log-likelihood comparison of models with and without PAIR as a predictor shows that the inclusion of PAIR accounts for more variance (χ^2^ = 34.58, p ≤ 0.0001), supporting the dyad-specific adaptive nature of this response. Both behavioral measures, dwell time and pupil diameter, are consistent with predicted early behavioral increases for in-person face processing.

### 3.2 Electrocortical measures for live in-person and virtual face processes

**Fig. 3.**
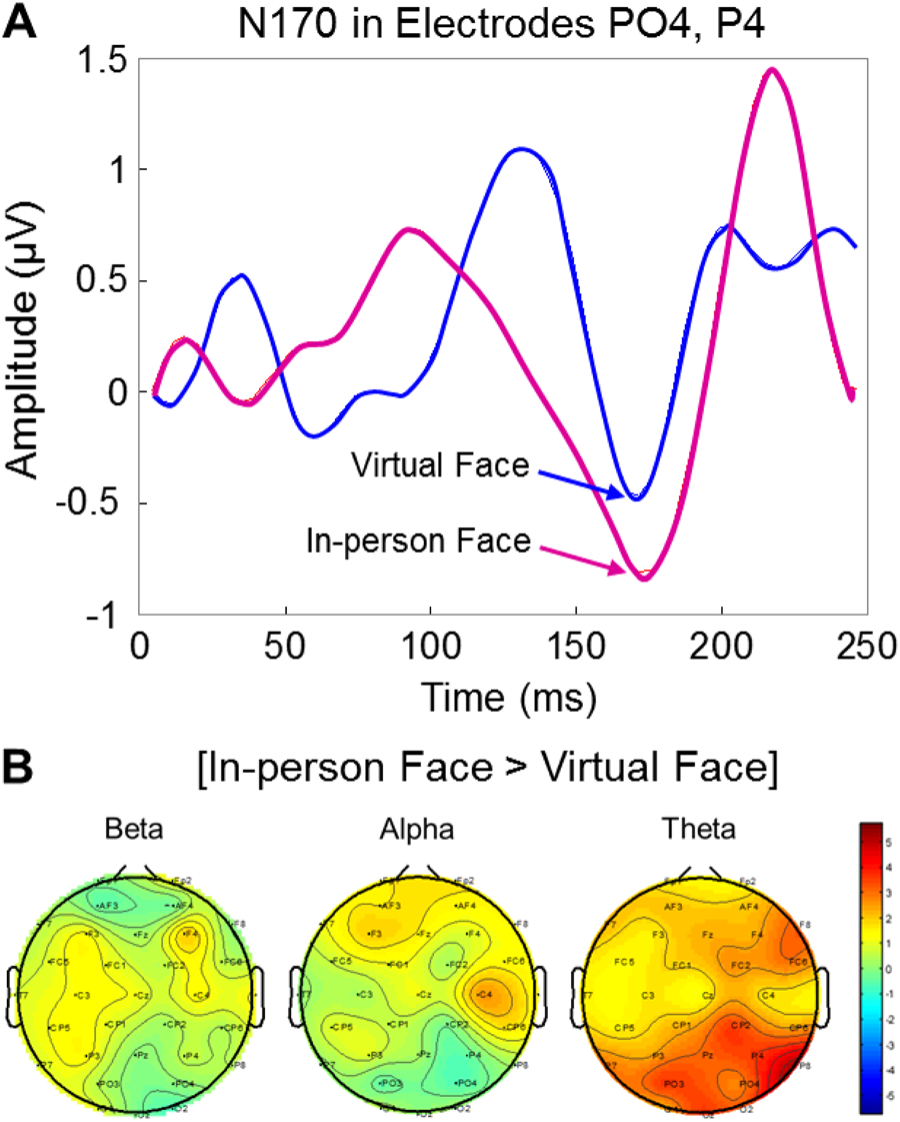
(A) N170 event-related potential is shown for In-person Face (red) and Virtual Face (blue) for electrodes PO4 (Extrastriate Visual Cortex and V3) and P4 (Supramarginal Gyrus and Somatosensory Association Cortex). The amplitudes of the signal at 170 ms are not statistically different. See Section 2 (Fig. 1D) for electrode configuration. Red: In-person Face, Blue: Virtual Face. (B) EEG signals within the first 250 ms were separated into frequency bands, including beta (13-30 Hz), alpha (8-13 Hz), and theta (4-8 Hz), using a wavelet decomposition algorithm. Topoplots display differences between frequency amplitudes for In-person vs. Virtual Face conditions determined by t-tests, as indicated on the color bar (range: -5 to +5). The theta band is increased for the In-person vs. Virtual Face condition (p ≤ 0.000015, t = 5.15).

Early visual sensing and pupil size increases (above) are consistent with the face-related averaged N170 event-related potentials, ERP, for In-person and Virtual faces detected by electrodes PO4 (Extrastriate Visual Cortex and V3) and P4 (Supramarginal Gyrus and Somatosensory Association Cortex). Both conditions, In-person (red) and Virtual (blue), produce the well-known N170 ERP signal during face viewing (Behrmann, Thomas, & Humphreys, 2006; Bentin et al., 1996; Corrigan et al., 2009; Deffke et al., 2007; Dravida, Ono, Noah, Zhang, & Hirsch, 2019; Naples, Wu, Mayes, & McPartland, 2017) at approximately 170 ms after the face onset (Fig. 3A). Separation of these signals into bandwidths indicates an increase in the theta band power spectrum (4-8 Hz) for the In-person condition relative to the Virtual condition (p ≤ 0.000015, t = 5.15) (Fig. 3B). No differences were observed in the beta and alpha spectra.

### 3.3 Hemodynamic measures for live in-person and virtual face processes

**Fig. 4.**
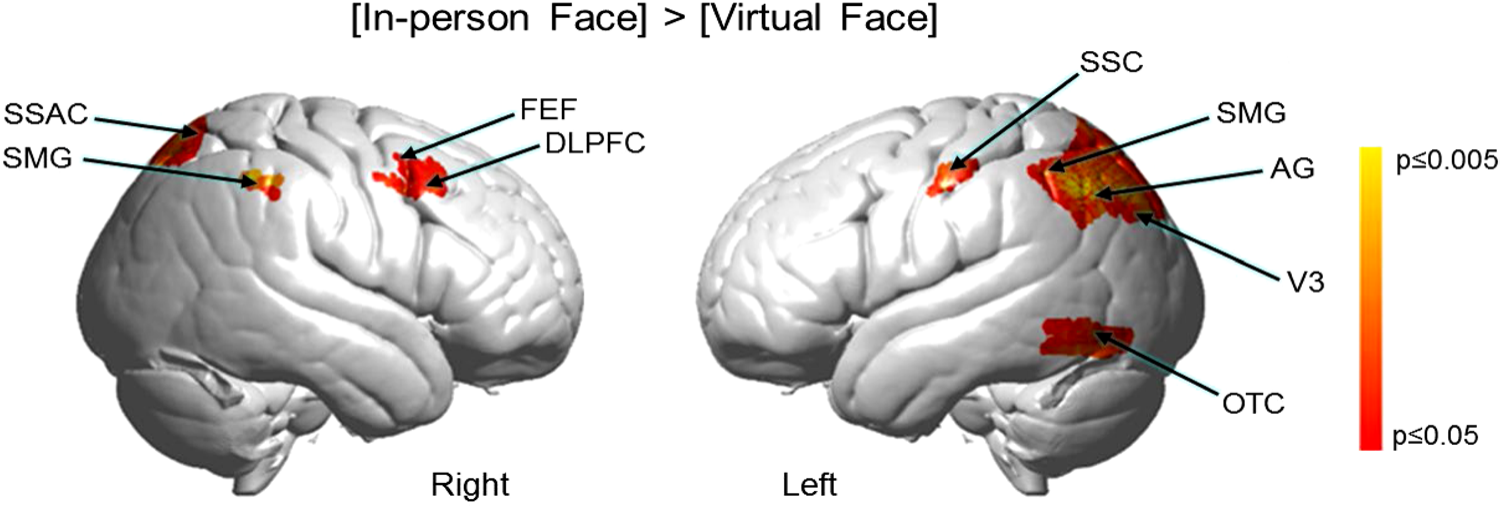
Contrast comparisons [In-person Face] > [Virtual Face] of the same partner based on the combined (Hb diff) OxyHb and deOxyHb signals (p ≤ 0.05). Activity is observed bilaterally in supramarginal gyrus (SMG); somatosensory association cortex (SSAC); frontal eye fields (FEF); and dorsolateral prefrontal cortex (DLPFC). Left hemisphere activity: SMG; angular gyrus (AG); occipitotemporal cortex (OTC); visual cortex (V3); and primary somatosensory cortex (SSC). See Supplementary Table S4. Note: Similar findings are also observed for the OxyHb signals (See Supplementary Fig. S1 and Supplementary Table S5) and the deOxyHb signal (See Supplementary Fig. S2 and Supplementary Table S6) in accordance with established best practices for fNIRS findings (Yücel et al., 2021). Not corrected for multiple comparisons.

Previous findings of live face gaze compared to simulated face gaze include activity in right dorsal stream (Hirsch et al., 2022; Kelley et al., 2021; Noah et al., 2020) and predict similar findings for this comparison based on the hypothesis that a real and present face is more salient than a real and virtual face. The contrast [In-person Face > Virtual Face] (Fig. 4) shows this predicted region-of-interest, ROI, activity in the dorsal stream located in the following clusters (p ≤ 0.05): right supramarginal gyrus (rSMG) (peak t = 2.66, df = 27, p < 0.0065) peak MNI coordinate of (66, −44, 48); somatosensory association cortex (SSAC) (peak t = 2.49, df = 27, p < 0.0096) peak MNI coordinate of (24, −66, 54); and frontal eye fields (FEF) (peak t = 1.97, df = 27, p < 0.0296) peak voxel MNI coordinate of (46, 20, 42). Increased activity was also observed in the left hemisphere including supramarginal gyrus (SMG) and angular gyrus (AG) (n of voxels = 851, peak t = 3.79, df = 27, (p < 0.0004), peak MNI coordinate of (-54, -62, 44)); occipitotemporal cortex (OTC) (peak t = 2.33, df = 27, (p < 0.0138) peak MNI coordinate of (-54, -56, -14)); and primary somatosensory cortex (SSC) (n of voxels = 44). See Supplementary Table S4.

### 3.4 Dyadic neural coupling

**Fig. 5.**
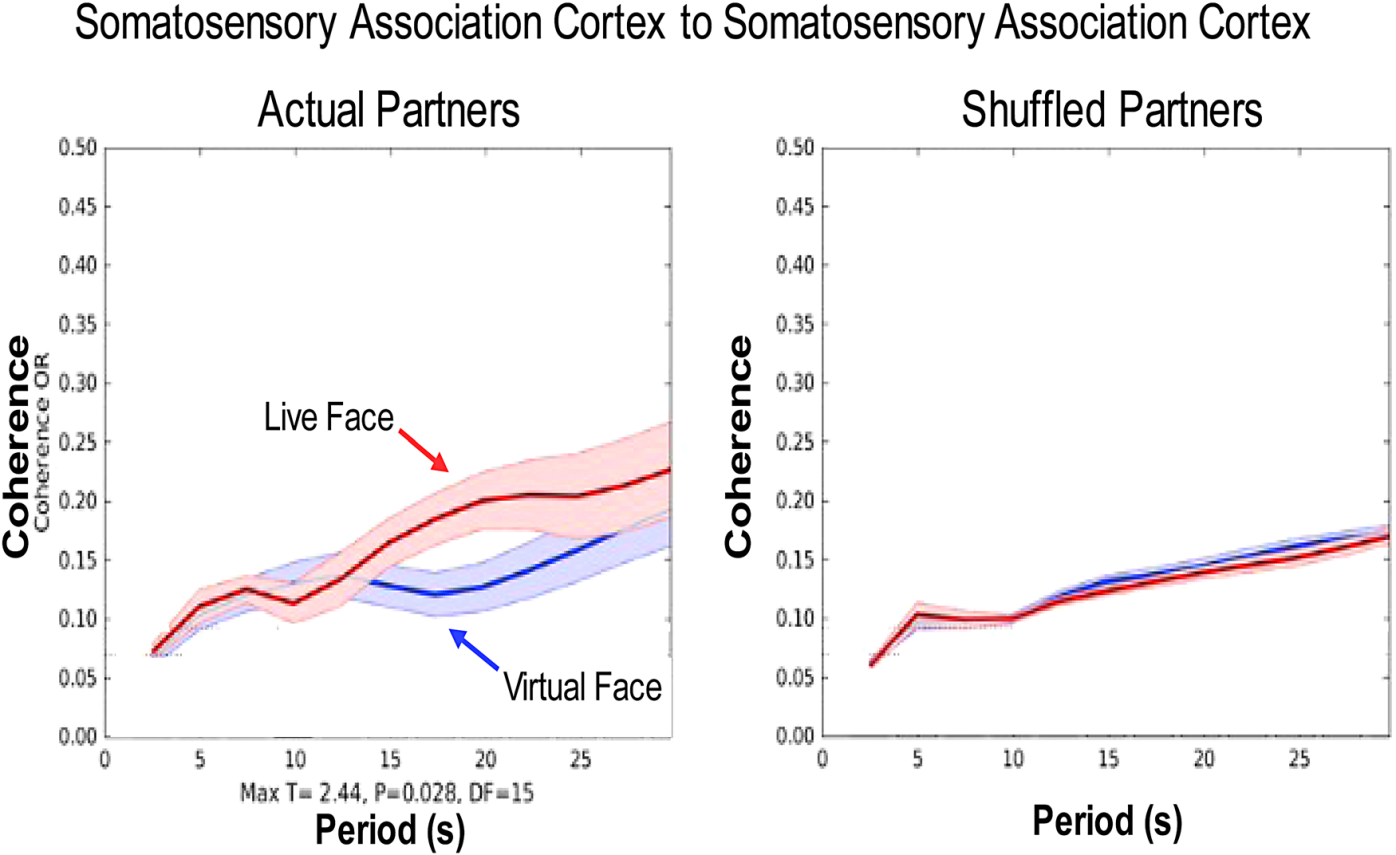
Cross-brain coherence between somatosensory cortices. Signal coherence between participants (y-axis) is plotted against the period of the frequency components (x-axis) for the In-person Face condition (red) and the Virtual Face condition (blue) (shaded areas: ±1 SEM) (p = 0.028). Left panel shows coherence between actual partners. Right panel shows coherence between shuffled partners. No significant effects were observed in shuffled partners. The comparison of actual and shuffled partners is consistent with the conclusion that coherence measures are sensitive to the reciprocal interactions between dyads. Note: an example of the more conventional format for coherence analysis is presented in Supplementary Figure S3 for a representative dyad.

Increased cross-brain coherence for the In-person condition (live real face) and Virtual condition (live, on-line, virtual face) is shown in Figure 5, left panel, between the somatosensory association cortices (SSAC) where there was increased activity for the In-person condition in the predicted ROI (Fig. 4). This region is part of the dorsal visual stream previously associated with salience detection (Gottlieb et al., 1998) and attention (Yantis, 1996) and previously reported in association with live face-to-face conditions (Hirsch et al., 2022). The temporal period of the signal wavelet (x-axis, seconds) and the average dyadic cross-brain coherence (y-axis, correlation coefficient) are shown for In-person Face (red) and Virtual Face (blue) conditions for signals located in dorsal somatosensory association cortices of the interacting brains (shaded areas: ±1 SEM) (p = 0.028). The observation that this coherence is greater than the coherence for the on-line condition guides our interpretation of the neural effects associated with in-person and on-line faces. The right panel shows the same data with the partners computationally exchanged or “shuffled” (i.e., participants are randomly assigned to dyads other than paired with their partners). This comparison eliminates the dyad-specific reciprocal interactive effects, i.e., the reciprocal dyadic behaviors are not present when the dyad pairs are shuffled. The overlapping functions in the right panel are consistent with the conclusion that the observed coherence effects between partners (left panel) are due to actual pair-specific shared social cues rather than task effects that are common to all conditions. These data suggest that the exchange of social cues is greater for the In-person condition and that these mechanisms are associated with dorsal stream activity.

## 4 Discussion

### 4.1 Real “in-person” vs. “on-line” face gaze

The human face is a highly salient and well-studied object category thought to be processed by functionally connected nodes within face-specialized complexes of the ventral stream including occipital, parietal, and temporal lobes (Arcaro & Livingstone, 2021; Diamond & Carey, 1986; Engell & Haxby, 2007; Haxby, Gobbini, Furey, Ishai, & Pietrini, 2001; Haxby, Gobbini, Furey, Ishai, Schouten, et al., 2001; Haxby et al., 2000; Ishai et al., 1999; Johnson et al., 2005; Kanwisher et al., 1997,^1998^; Tanaka & Farah, 1991). Accordingly, face-processing pathways are often assumed to include multiple regions with specializations for coding various aspects of face features (Chang & Tsao, 2017). However, this model is challenged to predict differences in visual pathways mediated by social context associated with the *actual presence* of a face vs. an on-line *representation* of the same actual face. In the case of this experiment, all social factors such as familiarity, gender subjective, biases, prior experience, associations, etc. were held constant since the partners were the same for both tasks, in-person and on-line. In addition to these common high-level social features, the live faces in both conditions shared common low-level facial features and differed only in the context of physical presence of the face even though the person was physically present in all conditions. Any observed differences raise impactful questions regarding the mechanisms of live social processes. Findings from this investigation suggest that differences occur at the visual sensing level (mean and standard variation of eye contact duration); the behavioral level (coherence and diameters of pupils); the electrocortical level (theta oscillations); the neuroimaging level (contrast between in-person and on-line faces); and the dyadic neural coupling level (coherence between neural signals in the dorsal parietal regions). Consistent with the constellation of these multi-modal findings, an increase in the neural coupling of the dorsal visual stream between somatosensory association cortices during in-person face processing suggests that the exchange of social cues is greater for the In-person condition and that these mechanisms are associated with dorsal stream activity. These multi-modal findings enrich the foundation for further development of dyadic models for face processing in live and natural conditions.

### 4.2 A multi-modal approach

Use of web conferencing platforms (e.g., Zoom, Skype, Teams, etc.) for conducting business as well as developing and maintaining interpersonal relationships has heightened awareness of possible differences between live in-person social encounters and live virtual encounters. Since the virtual encounters are primarily face-related, this raises the additional interesting question of whether or not the underlying face processing mechanisms differ depending upon the social context as represented by mode of presentation: live in-person or live on-line. A multi-modal approach was applied to address the multi-dimensional complexity of comparisons designed to simultaneously evaluate physiological, behavioral, and neural responses in pairs of interacting individuals as dyads in two conditions: live face-to-face gaze (in-person face) and live on-line face gaze (virtual face). Concurrent data recordings were acquired using functional near-infrared spectroscopy (fNIRS) for neuroimaging data, providing spatial maps of activity patterns and a measure of neural coupling between the interacting dyads, electroencephalography (EEG) for event-related potentials and temporal oscillation data, eye tracking including duration of eye contact, and pupil diameter for behavioral and physiological measures. A previously described two-person hyperscanning face-gaze paradigm (Hirsch et al., 2017, 2022; Kelley et al., 2021; Noah et al., 2020) was used to compare responses during in-person dyadic face gaze and with the same person in an on-line (Zoom-like) dyadic face-gaze task.

### 4.3 Separable pathways for live “in-person” and live “on-line” faces

The findings are consistent with separable neuroprocessing pathways for live faces presented in-person and for the same live faces presented over virtual media. First, at the visual acquisition level, longer dwell times on the face and reduced horizontal positional variation were observed for the live partner, suggesting that visual sensing mechanisms were more stable with longer durations between eye movements for live in-person faces. Pupil diameters were generally larger for in-person faces than for virtual faces, suggesting increased arousal for in-person faces; in addition, the magnitudes of the pupil responses were reciprocated by partners within dyads consistent with dyadic interactions. Both conditions produced the expected negative peak in the event-related EEG signal at approximately 170 ms after the stimulus onset, N170, which is a hallmark for early face processing and not expected to differ between these two conditions. Theta oscillations (4-8 Hz), previously associated with face processing (Balconi & Lucchiari, 2006; Dravida et al., 2019; Engell & Haxby, 2007; González-Roldan et al., 2011; Güntekin & Başar, 2014; G. Knyazev, Slobodskoj-Plusnin, & Bocharov, 2009; Miyakoshi, Kanayama, Iidaka, & Ohira, 2010; Pitcher, Dilks, Saxe, Triantafyllou, & Kanwisher, 2011; Zhang et al., 2012), were higher for the In-person Face condition, suggesting an early frequency band separation of live in-person face processes relative to live Virtual Face processes. Consistent with these visual sensing, behavioral, and electrocortical findings, neuroimaging findings indicated separable patterns of activity for the two conditions. Specifically, activity for the [In-person Face > Virtual on-line Face] contrast included increases in bilateral dorsal parietal regions. This divergence of pathways for live In-person vs. live Virtual on-line formats underscores the importance of ecological and social context in natural face processing.

### 4.4 Dorsolateral parietal brain regions and face processing

Ecological and social contexts include, for example, attention and saliency functions previously associated with the dorsal parietal regions based on electrophysiological recordings in the lateral intraparietal cortex (thought to be homologous to regions within the human dorsal parietal cortex) from awake and behaving monkeys (Fanini & Assad, 2009; Gottlieb et al., 1998). In these previous single-unit recordings of physiological processes, neural responses were observed when behaviorally significant stimuli appeared in receptive fields following naturally generated saccades and fixations. These same neurons were only weakly sensitive to ordinary and less salient objects that appeared in the same receptive fields. Dorsal parietal mechanisms in humans have also been shown to be selectively responsive to signals representing classes of stimuli that are salient, attention guided, and rewarding to the observer (Yantis, 1996). Accordingly, these findings of increased dorsal parietal activity during in-person face processing and the associated increased visual dwell times and pupil diameters provide concordant support for cooperative attentional, social salience, and visual sensing mechanisms linked to live in-person face processing and to associated activity in the dorsal visual stream. Increased neural coupling for the dorsal somatosensory association cortices between in-person dyads relative to webcam (virtual on-line) dyads further highlights a putative role for visual sensing, salience, and subtle micro-facial movements in face processing and the dynamic sharing of social cues.

### 4.5 “Zoom-like” technology and face processing

It is possible that detection of facial micromovements may be reduced with the virtual on-line format. Specifically, one hypothesis suggests that the dynamic social cues typically exchanged by interacting live faces are not similarly acquired or exchanged for the virtual on-line face. The shorter dwell times for the virtual condition may suggest that less information was conveyed by the oculomotor system. Further, the typically off-center and downward angle of the typical video camera gives a distorted view of a partner’s eyes in the virtual condition that may reduce activity in interactive and social processing streams. Face-to-face encounters that occur naturally are direct line-of-sight eye contacts, but this is not supported by current webcam technology. Although faces are viewed with high resolution, eye-to-eye interactions may be compromised or distorted due to the camera angles, and it is not possible for an individual to directly reciprocate eye contact when looking at a partner’s face on the screen: On the one hand, if a participant looks at the camera so that their partner can see their eyes, they can no longer focus on the screen and specifically on their partner’s eyes. On the other hand, if they focus on the screen when the webcam is located above the screen, it appears to their partner that they are looking below their direct line of sight. These technological considerations that distinguish between the in-person and virtual visual faces may be related to the observed differences between the two presentation formats, and suggest future directions for investigation of mechanisms that underlie live face processes.

### 4.6 Conclusion

Recent global adaptations to enforced social isolation due to the COVID-19 pandemic have led to the development of and dependence upon webcam on-line formats for live communications. The rapid and widespread use of this technology sets the stage for this timely question of how social interactions based on face gaze differ between live “in-person” and live virtual “on-line” (webcam) modes of presentation. The question also has scientific merit for its potential to advance understanding of face encoding pathways in the human brain in natural and spontaneous real-world circumstances. Feature-selective models are challenged to predict neural, behavioral, or physiological differences in live face-encoding pathways due to the consistency of facial and social features in both modes of presentation. Based on a novel multi-modal dyadic paradigm, we report increases in neural activity within the dorsal visual stream, increases in neural coupling as measured by cross-brain coherence, changes in visual sensing, increases in arousal as indicated by variations in pupil diameter, and increases in electrocortical responses in the theta band for live “in-person” face presentations relative to the same faces in virtual “Zoom-like” on-line mode. These findings underscore the significance of real faces and natural stimuli for investigations of live face processing and social interactions (Park et al., 2022), and highlight opportunities for the development of novel dynamical systems for investigation of real-time interactions between humans and virtual partners aimed at understanding mechanisms of behavior and neural coupling (Kelso, de Guzman, Reveley, & Tognoli, 2009).

## Supplementary Material

Supplementary Material

## Data Code and Availability Statement

The dataset has been submitted to Dryad in preparation for public access and has been assigned doi:10.5061/dryad.zpc866tct. It will be live as soon as the findings are published.
