# Supplementary material for: Separable processes for live “in-person” and live “zoom-like” faces
Source: Imaging Neurosci (Camb). 2023 Nov 7;1:imag-1-00027. doi: 10.1162/imag_a_00027 (PMC12007548; doi:10.1162/imag_a_00027)
Supplement: Supplementary Material [file imag_a_00027-supp.pdf]

Supplementary Information

Separable Neural and Visual Processes for Live "In-Person" and "Zoom-like" Faces

Nan Zhao[1,2], Xian Zhang[1], J. Adam Noah[1], Mark Tiede[1], and *Joy Hirsch[1,3,4,5,6]

Affiliations:

[1]Department of Psychiatry, Yale School of Medicine, New Haven, CT, 06511, United States

[2]School of Psychology and Cognitive Science, East China Normal University, Shanghai, China

[3]Department of Neuroscience, Yale School of Medicine, New Haven, CT, 06511, United States

[4]Department of Comparative Medicine, Yale School of Medicine, New Haven, CT, 06511, United States

[5]Wu Tsai Institute, Yale University, New Haven, CT 06511 United States

[6]Department of Medical Physics and Biomedical Engineering, University College London, London, United Kingdom

***Corresponding Author:**

Joy Hirsch, PhD
Yale School of Medicine
300 George St., Suite 902
New Haven, CT 06511
E-mail: joy.hirsch@yale.edu

Table S1a. fNIRS Channel identifier (number), group median coordinates (x,y,z) for anatomical regions, atlas-based probabilities for each channel Brodmann's Area, BA, and anatomical region. Montreal Neurological Institute (MNI) coordinates for each of the 60 channels per participant were determined by digitizing the locations of the optodes in relation to the 10-20 system based on conventional landmarks. See Figure 1D in the main text.

Table S1a Functional near infrared spectroscopy

Channel Number	MNI Coordinates[1] X	Y	Z	Probability	BA[2]	Anatomical Region
1	-42	-64	57	0.6	7	Somatosensory Association Cortex
				0.4	40	Supramarginal gyrus part of Wernicke's area
2	-53	-55	54	1	40	Supramarginal gyrus part of Wernicke's area
3	-48	-75	45	0.55	39	Angular gyrus; part of Wernicke's area
				0.24	19	V3
				0.16	7	Somatosensory Association Cortex
4	-51	25	36	0.7	9	Dorsolateral prefrontal cortex
				0.23	46	Dorsolateral prefrontal cortex
5	-60	-2	41	0.98	6	PreMotor and Supplementary Motor Cortex
6	-62	-26	47	0.37	2	Primary Somatosensory Cortex
				0.21	40	Supramarginal gyrus part of Wernicke's area
				0.2	1	Primary Somatosensory Cortex
7	-61	-46	47	1	40	Supramarginal gyrus part of Wernicke's area
8	-55	-67	42	0.67	39	Angular gyrus; part of Wernicke's area
				0.33	40	Supramarginal gyrus part of Wernicke's area
9	-50	36	25	0.99	46	Dorsolateral prefrontal cortex
10	-61	10	25	0.38	9	Dorsolateral prefrontal cortex
				0.23	44	pars opercularis part of Broca's area
				0.23	6	Pre-Motor and Supplementary Motor Cortex
				0.17	45	pars triangularis Broca's area
11	-66	-15	34	0.36	6	PreMotor and Supplementary Motor Cortex
				0.2	1	Primary Somatosensory Cortex
				0.2	3	Primary Somatosensory Cortex
12	-66	-36	41	0.91	40	Supramarginal gyrus part of Wernicke's area
13	-61	-58	35	0.68	40	Supramarginal gyrus part of Wernicke's area
				0.32	39	Angular gyrus part of Wernicke's area
14	-47	-84	29	0.53	39	Angular gyrus; part of Wernicke's area
				0.47	19	V3
15	-59	22	14	0.69	45	pars triangularis Broca's area
				0.26	44	pars opercularis part of Broca's area
16	-66	-4	20	0.47	6	PreMotor and Supplementary Motor Cortex
				0.3	43	Subcentral area
17	-69	-27	28	0.6	40	Supramarginal gyrus part of Wernicke's area
				0.23	2	Primary Somatosensory Cortex
18	-67	-48	25	0.7	40	Supramarginal gyrus part of Wernicke's area
				0.3	22	Superior Temporal Gyrus

19	-53	-76	20	0.56	39	Angular gyrus; part of Wernicke's area
				0.44	19	V3
20	-55	34	5	0.43	45	pars triangularis Broca's area
				0.3	47	Inferior prefrontal gyrus
				0.24	46	Dorsolateral prefrontal cortex
21	-63	5	-1	0.61	22	Superior Temporal Gyrus
				0.23	21	Middle Temporal gyrus
22	-69	-16	9	0.48	42	Primary and Auditory Association Cortex
				0.38	22	Superior Temporal Gyrus
23	-69	-39	13	0.92	22	Superior Temporal Gyrus
24	-64	-60	8	0.48	21	Middle Temporal gyrus
				0.29	22	Superior Temporal Gyrus
25	-48	-86	10	0.88	19	V3
26	-58	14	-9	0.48	38	Temporopolar area
				0.26	22	Superior Temporal Gyrus
27	-68	-9	-11	1	21	Middle Temporal gyrus
28	-71	-29	-5	0.87	21	Middle Temporal gyrus
29	-68	-50	-3	0.72	21	Middle Temporal gyrus
30	-59	-70	-1	0.53	37	Fusiform gyrus
				0.39	19	V3
31	47	-61	55	0.59	40	Supramarginal gyrus part of Wernicke's area
				0.41	7	Somatosensory Association Cortex
32	50	-71	39	0.78	39	Angular gyrus; part of Wernicke's area
				0.17	19	V3
33	55	-53	53	1	40	Supramarginal gyrus part of Wernicke's area
34	58	-63	36	0.56	39	Angular gyrus; part of Wernicke's area
				0.44	40	Supramarginal gyrus part of Wernicke's area
35	63	-42	48	1	40	Supramarginal gyrus part of Wernicke's area
36	64	-18	47	0.33	6	PreMotor and Supplementary Motor Cortex
				0.22	3	Primary Somatosensory Cortex
				0.21	1	Primary Somatosensory Cortex
37	61	5	38	0.63	6	PreMotor and Supplementary Motor Cortex
				0.34	9	Dorsolateral prefrontal cortex
38	53	29	34	0.58	9	Dorsolateral prefrontal cortex
				0.42	46	Dorsolateral prefrontal cortex
39	49	-81	19	0.73	19	V3
				0.27	39	Angular gyrus part of Wernicke's area
40	64	-54	34	0.92	40	Supramarginal gyrus part of Wernicke's area
41	69	-31	39	0.67	40	Supramarginal gyrus part of Wernicke's area
				0.17	2	Primary Somatosensory Cortex
				0.16	1	Primary Somatosensory Cortex
42	67	-6	33	0.88	6	PreMotor and Supplementary Motor Cortex
43	62	13	22	0.34	44	pars opercularis; part of Broca's area
				0.32	9	Dorsolateral prefrontal cortex
				0.27	45	pars triangularis Broca's area

44	52	39	24	0.95	46	Dorsolateral prefrontal cortex
45	56	-74	17	0.61	39	Angular gyrus; part of Wernicke's area
				0.39	19	V3
46	69	-43	25	0.68	40	Supramarginal gyrus part of Wernicke's area
				0.28	22	Superior Temporal Gyrus
47	70	-19	25	0.3	40	Supramarginal gyrus part of Wernicke's area
				0.23	43	Subcentral area
				0.17	2	Primary Somatosensory Cortex
48	68	0	17	0.55	6	PreMotor and Supplementary Motor Cortex
				0.17	22	Superior Temporal Gyrus
49	60	26	13	0.74	45	pars triangularis Broca's area
				0.2	46	Dorsolateral prefrontal cortex
50	48	-84	4	0.79	19	V3
				0.21	18	Visual Association Cortex (V2)
51	66	-55	8	0.5	21	Middle Temporal gyrus
				0.43	22	Superior Temporal Gyrus
52	72	-34	12	0.67	22	Superior Temporal Gyrus
				0.32	42	Primary and Auditory Association Cortex
53	70	-11	6	0.46	22	Superior Temporal Gyrus
				0.3	42	Primary and Auditory Association Cortex
				0.18	21	Middle Temporal gyrus
54	64	5	-1	0.6	22	Superior Temporal Gyrus
				0.26	21	Middle Temporal gyrus
55	56	36	3	0.47	47	Inferior prefrontal gyrus
				0.31	45	pars triangularis Broca's area
				0.18	46	Dorsolateral prefrontal cortex
56	59	-67	-3	0.59	37	Fusiform gyrus
				0.34	19	V3
57	70	-45	-2	0.68	21	Middle Temporal gyrus
				0.24	22	Superior Temporal Gyrus
58	73	-24	-5	0.76	21	Middle Temporal gyrus
				0.24	22	Superior Temporal Gyrus
59	68	-6	-14	1	21	Middle Temporal gyrus
60	59	17	-10	0.62	38	Temporopolar area
				0.24	47	Inferior prefrontal gyrus

Table S1b. Electrode identifier (Name), group median coordinates (x,y,z) for anatomical regions, atlas-based probabilities for each location, Brodmann's Area, BA, and anatomical region. Montreal Neurological Institute (MNI) coordinates for each of the 32 electrodes per participant were determined by digitizing the locations of the electrodes in relation to the 10-20 system based on conventional landmarks. See Figure 1D in the main text.

Table S1b Electroencephalography

	MNI Coordinates[1]					
**Name**	**X**	**Y**	**Z**	**Probability**	**BA[2]**	**Anatomical Region**
fp1	-27	64	19	1	10	Frontopolar area
fp2	27	66	18	1	10	Frontopolar area
af3	-26	49	41	0.78	9	Dorsolateral prefrontal cortex
				0.22	8	Includes Frontal eye fields
af4	25	52	41	0.78	9	Dorsolateral prefrontal cortex
				0.22	8	Includes Frontal eye fields
f7	-57	22	20	0.58	45	pars triangularis Broca's area
				0.16	44	pars opercularis part of Broca's area
				0.16	46	Dorsolateral prefrontal cortex
f3	-45	25	46	0.7	8	Includes Frontal eye fields
				0.3	9	Dorsolateral prefrontal cortex
fz	-1	33	59	0.58	8	Includes Frontal eye fields
				0.42	6	Pre-Motor and Supplementary Motor Cortex
f4	45	31	44	0.53	8	Includes Frontal eye fields
				0.47	9	Dorsolateral prefrontal cortex
f8	59	27	13	0.74	45	pars triangularis Broca's area
				0.24	46	Dorsolateral prefrontal cortex
fc5	-66	-3	22	0.56	6	PreMotor and Supplementary Motor Cortex
				0.26	43	Subcentral area
fc1	-25	8	69	1	6	PreMotor and Supplementary Motor Cortex
fc2	23	10	70	1	6	PreMotor and Supplementary Motor Cortex
fc6	67	2	16	0.53	6	PreMotor and Supplementary Motor Cortex
				0.16	44	pars opercularis part of Broca's area
t7	-71	-29	1	0.51	21	Middle Temporal gyrus
				0.31	22	Superior Temporal Gyrus
				0.17	42	Primary and Auditory Association Cortex
c3	-61	-23	49	0.37	2	Primary Somatosensory Cortex
				0.26	1	Primary Somatosensory Cortex
c4	64	-20	46	0.28	1	Primary Somatosensory Cortex
				0.26	6	Pre-Motor and Supplementary Motor Cortex
				0.18	3	Primary Somatosensory Cortex
				0.17	2	Primary Somatosensory Cortex
t8	72	-23	-5	0.72	21	Middle Temporal gyrus
				0.28	22	Superior Temporal Gyrus
cp5	-66	-47	31	0.98	40	Supramarginal gyrus part of Wernicke's area
cp1	-22	-39	76	0.41	3	Primary Somatosensory Cortex
				0.27	5	Somatosensory Association Cortex

				0.16	2	Primary Somatosensory Cortex
cp2	25	-38	76	0.41	3	Primary Somatosensory Cortex
				0.25	5	Somatosensory Association Cortex
				0.16	4	Primary Motor Cortex
				0.15	2	Primary Somatosensory Cortex
cp6	69	-43	25	0.68	40	Supramarginal gyrus part of Wernicke's area
				0.3	22	Superior Temporal Gyrus
p7	-58	-71	5	0.42	37	Fusiform gyrus
				0.38	19	V3
				0.17	39	Angular gyrus part of Wernicke's area
p3	-41	-61	60	0.57	7	Somatosensory Association Cortex
				0.42	40	Supramarginal gyrus part of Wernicke's area
pz	1	-72	61	1	7	Somatosensory Association Cortex
p4	45	-61	57	0.52	7	Somatosensory Association Cortex
				0.48	40	Supramarginal gyrus part of Wernicke's area
p8	59	-68	-3	0.56	37	Fusiform gyrus
				0.39	19	V3
po3	-22	-91	37	1	19	V3
po4	26	-90	35	1	19	V3
o1	-22	-102	-5	0.99	18	Visual Association Cortex (V2)
oz	0	-102	0	0.83	18	Visual Association Cortex (V2)
				0.17	17	Primary Visual Cortex (V1)
o2	24	-101	-5	0.96	18	Visual Association Cortex (V2)

Supplementary Table S2. Experiment ID; age range; gender; handedness; race; and racial composition of dyads.

ID	Age range	Female	Male	Other	RT handed?	Asian	White	Latina	Biracial	Dyad
01A	40-45	1	0	0	Yes	0	0	0	1	Biracial & White
01B	20-25	1	0	0	Yes	0	1	0	0	
02A	60-65	0	1	0	Yes	0	1	0	0	White & White
02B	30-35	0	1	0	Yes	0	1	0	0	
03A	20-25	0	1	0	Yes	0	1	0	0	White & White
03B	25-30	1	0	0	No	0	1	0	0	
04A	20-25	0	1	0	Yes	0	1	0	0	White & White
04B	25-30	1	0	0	Yes	0	1	0	0	
05A	45-50	1	0	0	Yes	0	0	1	0	Latina & Asian
05B	25-30	0	1	0	Yes	1	0	0	0	
06A	30-35	0	1	0	Yes	0	1	0	0	Asian & White
06B	30-35	1	0	0	Yes	1	0	0	0	
07A	30-35	1	0	0	Yes	0	0	1	0	Latina & White
07B	25-30	1	0	0	No	0	1	0	0	
08A	20-25	1	0	0	Yes	1	0	0	0	Asian & Asian
08B	20-25	1	0	0	Yes	1	0	0	0	
09A	20-25	1	0	0	Yes	1	0	0	0	Asian & Asian
09B	20-25	1	0	0	Yes	1	0	0	0	
10A	20-25	1	0	0	Yes	0	1	0	0	White & White
10B	25-30	1	0	0	Yes	0	1	0	0	
11A	20-25	0	1	0	Yes	1	0	0	0	Asian & White
11B	20-25	0	0	1	Yes	0	1	0	0	
12A	30-35	0	1	0	Yes	1	0	0	0	Asian & Asian
12B	25-30	1	0	0	Yes	1	0	0	0	
13A	35-40	0	1	0	Yes	0	1	0	0	White & White
13B	35-40	0	1	0	Yes	0	1	0	0	
14A	20-25	1	0	0	Yes	1	0	0	0	Asian & White
14B	20-25	1	0	0	Yes	1	0	0	0	
		17	10	1	26 R<br>2 L	11	14	2	1	

Supplementary Table S3. Demographics summary table for information presented in Supplementary Table S1.

Supplementary Table S3

**Demographics summary**		
**Gender**	**n**	**%**
Female	17	61%
Male	10	36%
Other*	1	4%
	**28**	100%
**Dyad gender**		
Female & Female	6	
Female & Male	5	
Male & Male	2	
Male & Other	1	
**Race**		
Asian	14	
White	11	
Latina	2	
Biracial	1	
**Dyad race**		
White & White	5	
Asian & Asian	4	
Asian & White	2	
Latina & White	1	
Latina & Asian	1	
Biracial & White	1	
**Age (years)**		
Mean	28.4	
Median	26	
Range	18-63	
SD	± 9.8	
**Handedness**		
Right	26	
Left	2	

*Other: self-identified gender is not male or female

Supplementary Table S4. GLM Contrast comparison:
[In-person Face > Virtual Face] (deOxyHb + OxyHb signals)

Contrast	Contrast Threshold	Peak Voxels						Anatomical Regions in Cluster	BA[3]	Prob.[4]	n of Voxels[5]
		MNI Coordinates[1]			t value	p	df[2]				
**[In-person Face > Virtual Face]**	p = 0.05	-54	-62	44	3.79	0.001	27	Supramarginal Gyrus	40	0.61	851
								Angular Gyrus	39	0.35	
		-54	-56	-14	2.33	0.014	27	Occipitotemporal Cortex	37	0.63	103
								Inferior Temporal Gyrus	20	0.19	
								Extrastriate Visual Cortex (V3)	19	0.15	
		-58	-14	44	2.34	0.013	27	Pre- and Supplementary Motor Cortex	6	0.50	44
								Primary Somatosensory Cortex	3	0.19	
								Primary Somatosensory Cortex	1	0.13	
		46	20	42	1.97	0.030	27	Frontal Eye Fields	8	0.51	88
								Dorsolateral Prefrontal Cortex	9	0.42	
		24	-66	54	2.49	0.010	27	Somatosensory Association Cortex	7	1.00	46
		60	-44	48	2.66	0.006	27	Supramarginal Gyrus	40	0.98	10

[1]Coordinates are based on the MNI system and (-) indicates left hemisphere. [2]df = degrees of freedom. [3]BA = Brodmann Area. [4]Probability of inclusion in cluster. [5]"n of Voxels" refers to a relative index of cluster size on the rendered brain

[In-person Face] > [Virtual Face], OxyHb Signals

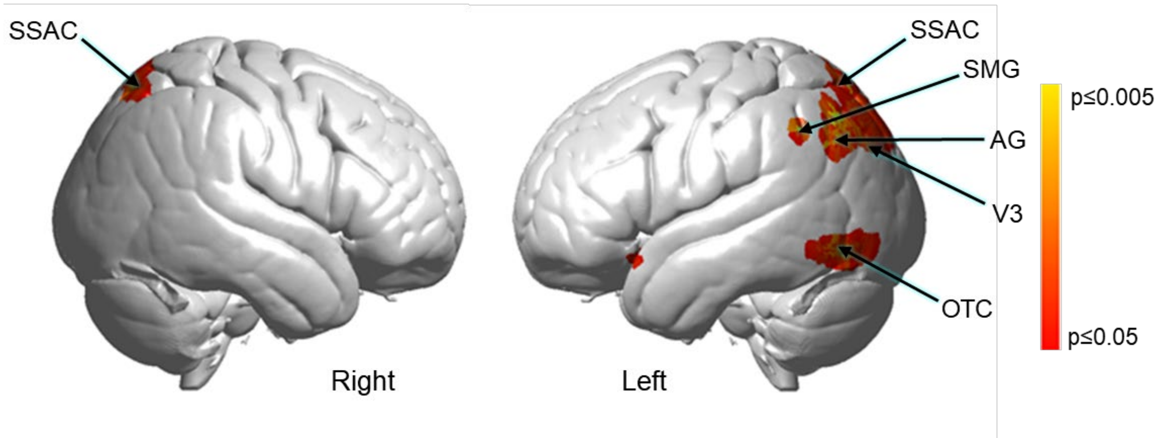

Supplementary Figure S1. GLM Contrast comparison [In-person Face > Virtual Face] based on oxyhemoglobin (OxyHb) signals. See Table S5.

Supplementary Table S5. GLM Contrast comparison: [In-person Face > Virtual Face] (OxyHb signals)

Contrast	Contrast Threshold	Peak Voxels MNI Coordinates[1]			t value	p	df[2]	Anatomical Regions in Cluster	BA[3]	Prob.[4]	n of Voxels[5]
**[In-person Face > Virtual Face]**	p = 0.05	-46	-64	48	3.09	0.002	27	Supramarginal Gyrus	40	0.44	321
								Somatosensory Association Cortex	7	0.28	
								Angular Gyrus	39	0.24	
		-48	-62	-12	2.48	0.010	27	Extrastriate Visual Cortex (V3)	19	0.58	161
								Occipitotemporal Cortex	37	0.36	
		-44	16	-14	1.87	0.036	27	Temporopolar Area	38	0.74	12
								Inferior Frontal Gyrus	47	0.25	
		-24	-62	58	2.06	0.025	27	Somatosensory Association Cortex	7	1.00	12
		-60	-50	40	2.89	0.004	27	Supramarginal Gyrus	40	0.95	10
		26	-66	54	2.33	0.014	27	Somatosensory Association Cortex	7	1.00	20

[1]Coordinates are based on the MNI system and (-) indicates left hemisphere. [2]df = degrees of freedom. [3]BA = Brodmann Area. [4]Probability of inclusion in cluster.
[5]"n of Voxels" refers to a relative index of cluster size on the rendered brain

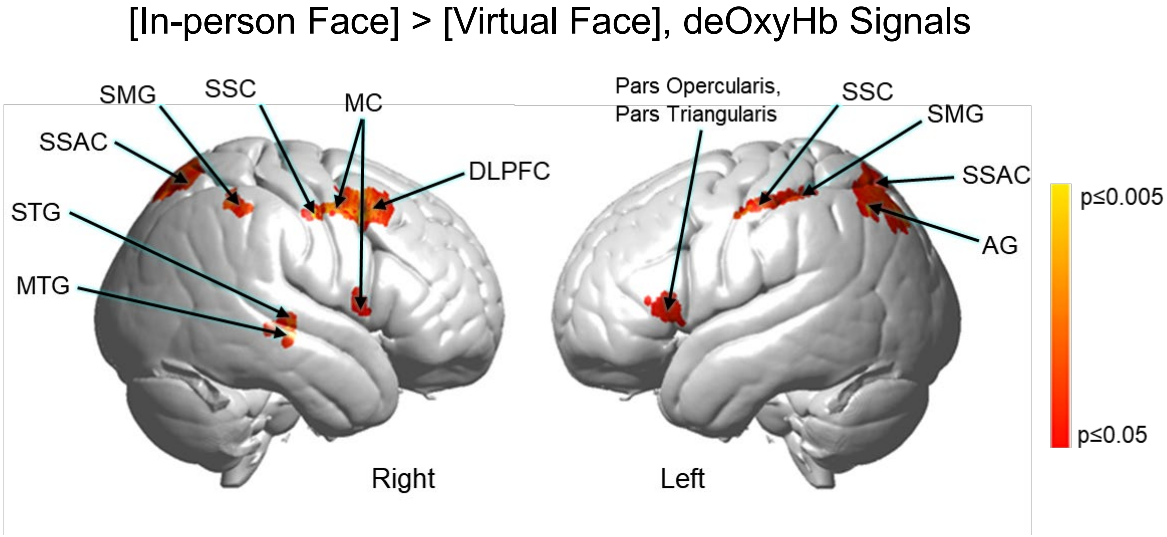

Supplementary Figure S2. GLM Contrast comparison [In-person Face > Virtual Face] based on deoxyhemoglobin (deOxyHb) signals. See Table S6.

Supplementary Table S6. GLM Contrast comparison: [In-person Face > Virtual Face] (deOxyHb signals)

Contrast	Contrast Threshold	Peak Voxels MNI Coordinates[1]			t value	p	df[2]	Anatomical Regions in Cluster	BA[3]	Prob.[4]	n of Voxels[5]
**[In-person Face > Virtual Face]**	p = 0.05	-48	-66	50	2.41	0.011	27	Supramarginal Gyrus	40	0.43	104
								Somatosensory Association Cortex	7	0.28	
								Angular Gyrus	39	0.23	
		-52	18	2	1.93	0.032	27	Inferior Frontal Gyrus	47	0.29	62
								Pars Triangularis	45	0.27	
								Superior Temporal Gyrus	22	0.18	
								Pars Opercularis	44	0.13	
								Temporopolar Area	38	0.13	
		-52	-22	48	2.07	0.024	27	Primary Somatosensory Cortex	3	0.24	37
								Primary Somatosensory Cortex	2	0.24	
								Primary Somatosensory Cortex	1	0.21	
								Pre- and Supplementary Motor Cortex	6	0.15	
		58	-10	48	2.64	0.007	27	Pre- and Supplementary Motor Cortex	6	0.62	229
								Primary Somatosensory Cortex	3	0.17	
								Primary Motor Cortex	4	0.11	
		20	-62	56	2.37	0.013	27	Somatosensory Association Cortex	7	1.00	40
		62	8	8	1.89	0.035	27	Superior Temporal Gyrus	22	0.37	28
								Pars Opercularis	44	0.26	
								Pre- and Supplementary Motor Cortex	6	0.18	
								Pars Triangularis	45	0.12	
		64	-20	-2	2.37	0.013	27	Middle Temporal Gyrus	21	0.58	17
								Superior Temporal Gyrus	22	0.26	
								Auditory Primary and Association Cortex	42	0.16	

[1]Coordinates are based on the MNI system and (-) indicates left hemisphere. [2]df = degrees of freedom. [3]BA = Brodmann Area. [4]Probability of inclusion in cluster. [5]"n of Voxels" refers to a relative index of cluster size on the rendered brain

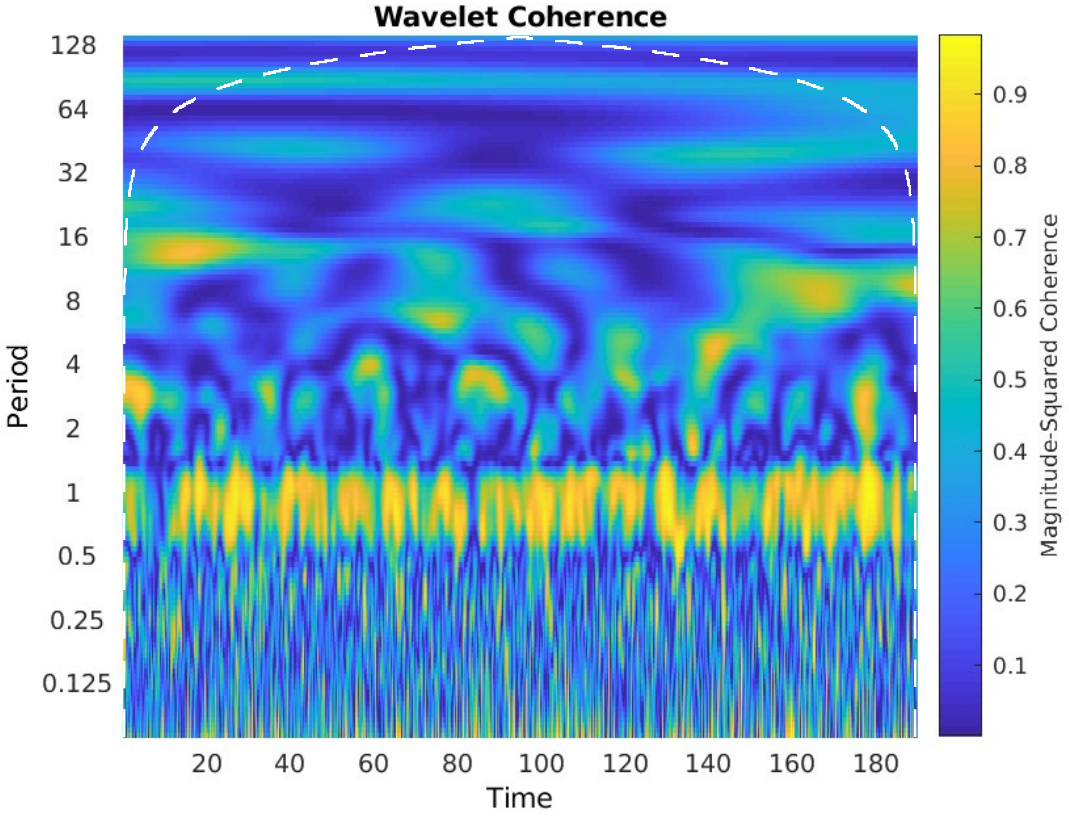

Supplementary Fig S3. An illustration of a standard wavelet coherence plot for channel 6 of a representative pair of subjects. The units of both the period (y-axis) and the time (x-axis) are seconds. The color-bar on the right indicates the coherence, i.e the correlation between partners. The data represent residual neural signals(K. J. Friston et al., 1997). The yellow band centered at period 1.0 is consistent with cardiac signals, and at approximately 3.0 is consistent with the experimental interval. The white dashed line represents the COI (cone of influence). Data outside the COI line are invalid.
